# Oxyresveratrol as a novel ferroptosis inducer exhibits anticancer activity against breast cancer via the EGFR/PI3K/AKT/GPX4 signalling axis

**DOI:** 10.3389/fphar.2024.1527286

**Published:** 2025-01-15

**Authors:** Lei Xiang, Qingzhou Li, Zhiwei Guan, Guilin Wang, Xiankuo Yu, Xianwen Zhang, Guochen Zhang, Jushan Hu, Xue Yang, Mingrui Li, Xilinqiqige Bao, Yumei Wang, Dong Wang

**Affiliations:** ^1^ School of Basic Medical Sciences, State Key Laboratory of Southwestern Chinese Medicine Resources, Chengdu University of Traditional Chinese Medicine, Chengdu, China; ^2^ School of Pharmacy, Chengdu University of Traditional Chinese Medicine, Chengdu, China; ^3^ School of Clinical Medicine, Chengdu University of Traditional Chinese Medicine, Chengdu, China; ^4^ School of Medical and Life Sciences, Chengdu University of Traditional Chinese Medicine, Chengdu, China; ^5^ Medical Innovation Center for Nationalities, Inner Mongolia Medical University, Hohhot, China

**Keywords:** oxyresveratrol, breast cancer, HTS^2^, gene expression profiles, ferroptosis, GPX4, EGFR/PI3K/AKT

## Abstract

**Introduction:**

Oxyresveratrol (ORes) exhibits significant anticancer activity, particularly against breast cancer. However, its exact mechanism of action (MOA) remains unclear. This study aimed to investigate the pharmacological activity and underlying MOA.

**Methods:**

The inhibitory effect of ORes on breast cancer cell growth was confirmed, and the effective concentrations were determined for further experiments. Gene expression profiles (GEPs) were collected from MDA-MB-231 cells treated with ORes at varying concentrations using HTS^2^. Bioinformatics tools were used to predict the anticancer activity and MOA of ORes. Ferroptosis markers (ferrous ions, reactive oxygen species, lipid peroxidation, and GPX4 expression) were assessed, and mitochondrial morphology was observed. The effect of ORes on tumour growth was evaluated *in vivo*, along with the analysis of ferroptosis in tissues. The MOA was explored using L1000, Drug Gene DataBase (DGDB), and Western blotting analyses.

**Results:**

ORes significantly reduces breast cancer cell viability and proliferation in a concentration-dependent manner, with IC_50_ values of 104.8 μM, 150.2 μM, and 143.6 μM in MDA-MB-231, BT-549, and 4T1 cells, respectively. GEPs induced by ORes were significantly enriched in the ferroptosis and PI3K/AKT signalling pathways. ORes inhibited breast cancer cell growth, increased intracellular ferrous ion levels, reactive oxygen species, and lipid peroxidation, and induced ferroptosis-related mitochondrial alterations. These effects were associated with decreased GPX4 expression and suppression of EGFR, phosphorylated PI3K, and phosphorylated AKT. ORes inhibited tumour growth, enhanced iron deposition, and reduced GPX4 expression in tumour tissues *in vivo*. Notably, treatment with the ferroptosis inhibitor ferrostatin-1 (Ferr-1) attenuated the anticancer effects of ORes, confirming the pivotal role of ferroptosis in ORes-mediated breast cancer inhibition.

**Conclusion:**

ORes inhibits breast cancer cell growth by inducing ferroptosis through suppression of the EGFR/PI3K/AKT/GPX4 signalling axis. This study suggests that ORes holds promise as a potential therapeutic agent for breast cancer and warrants further investigation into its clinical applications and potential integration into existing treatment regimens.

## 1 Introduction

Cancer is the second leading cause of death globally, claiming nearly eight million lives each year (Al-Warhi et al., 2023; Hu et al., 2024). Breast cancer is one of the most prevalent malignant tumours (Li Y. et al., 2023; Mou et al., 2024) and remains the leading cause of cancer-related mortality in women (Sung et al., 2021). Despite significant advances in early diagnosis and treatment, numerous challenges remain. Traditional approaches such as surgery, radiotherapy, and chemotherapy, although effective in some cases, often yield only limited success and are associated with severe side effects (Vagia et al., 2020). Although targeted therapies and immunotherapies have brought new hope, issues such as drug resistance and variability in patient responses continue to pose significant challenges (Song et al., 2020). Consequently, there is an urgent need to develop novel therapeutic agents and innovative treatment strategies to enhance the survival and quality of life of patients with breast cancer.

Natural products and their derivatives are rich sources for drug discovery (Xu et al., 2019). Natural products are widely used for the prevention and treatment of various diseases due to their significant pharmacological activity (Pandey et al., 2019), multi-target effects (Ryu et al., 2019), and low toxicity (Fan et al., 2021; Ahn et al., 2021). For example, artemisinin, discovered by the Nobel Prize winner Youyou Tu, has been successfully used to treat malaria (Su and Miller, 2015). Identifying the pharmacological activities and mechanisms of action (MOA) of natural products and their derivatives is crucial for enriching drug libraries, treating complex diseases, discovering new therapeutic targets, and advancing traditional medicine (Bhowmick et al., 2022).

Oxyresveratrol (trans-2,3′,4,5′-tetrahydroxystilbene, ORes) is a natural stilbene present in the heartwood of *Artocarpus lakoocha Roxb* (Choi et al., 2018; Dhakar et al., 2019). *Artocarpus lakoocha Roxb* is widely used in Southeast Asian countries to treat various ailments (Sonkar et al., 2015). A light brown powder obtained from the wood chips of *Artocarpus lakoocha Roxb* has traditionally been used to treat the intestinal fluke *Haplorchis taichui* (Wongsawad et al., 2005) and *taeniasis* (Charoenlarp et al., 1989). The pharmacokinetics of ORes have been investigated in both animal and human models. In rats, ORes is rapidly absorbed from the gastrointestinal tract, reaching peak plasma concentrations within 15 min, and is primarily excreted in the bile and urine as major metabolites, including glucuronides and sulfates (Qiu et al., 1996; Huang et al., 2008; Huang et al., 2009a; Huang et al., 2009b). In humans, ORes undergoes deglycosylation by intestinal bacteria, followed by hepatic conjugation to form ORes glucuronide and sulfate, with UDP-glucuronosyltransferases playing a key role in this process (Hu et al., 2014). Furthermore, studies have demonstrated that the oral bioavailability of ORes can be enhanced by co-administration with compounds such as piperine (Junsaeng et al., 2019). These findings highlight the rapid absorption, metabolism, and elimination of ORes, suggesting a favorable pharmacokinetic profile for oral administration and potential clinical applications.

ORes contains an additional hydroxyl group on the aromatic ring compared to resveratrol (trans-3,5,4′-trihydroxystilbene, Res) (Aggarwal et al., 2004; Chao et al., 2008). Res has been extensively studied and is known for its antioxidant (Meneses-Gutiérrez et al., 2019), anti-inflammatory (Avotri et al., 2019), cardiovascular protective (Matsumura et al., 2018), anticancer (Chang et al., 2018), and anti-aging effects (Okamoto et al., 2022). However, low oral bioavailability, rapid metabolism, and high clearance *in vivo* significantly limit its therapeutic efficacy (Peñalva et al., 2018). Its sensitivity to light, heat, and oxygen further compromises its stability and potency (Neves et al., 2016), restricting its clinical application. Although Res and ORes differ in their chemical structures and biological activities, they share similar effects in terms of antioxidant, anti-inflammatory, and cardiovascular protective effects and anticancer potential, including against breast cancer (Sr et al., 2014). Notably, ORes has demonstrated high bioactivity and stability (Dhakar et al., 2019), making them promising candidates for clinical use. ORes exhibits stronger antioxidant activity due to the electron delocalization effect of its 2-OH group, which enhances its bioactivity and results in superior neuroprotective and hepatoprotective effects (Chao et al., 2008; Apak et al., 2016; Shah et al., 2021). Furthermore, ORes forms a hydrogen bond with the Met280 residue of tyrosinase, enhancing its inhibitory activity against tyrosinase (Zeng et al., 2021). Studies have showed that ORes can inhibit cancer cell growth *in vitro* (Radapong et al., 2021), but the underlying anti-breast cancer effects of MOAs remain unclear. Therefore, further research is required to validate the pharmacological activity of ORes and elucidate their role in combating breast cancer.

Predicting the pharmacological activities and MOA of compounds using gene expression profiles (GEPs) is an innovative and effective method (Chen et al., 2020; Ahmed et al., 2022). GEPs offer a comprehensive snapshot of gene transcription in cells and tissues under various conditions (Hughes et al., 2000). Analysing these profiles not only reveals potential MOA but also identifies reliable candidate molecules for new drug development (Subramanian et al., 2017). With the advancement of high-throughput sequencing technologies and bioinformatics tools, the acquisition and processing of GEP data have become more efficient and accurate (Tian et al., 2023). High-throughput sequencing-based high-throughput screening (HTS^2^) is a powerful technology that enables the detection of thousands of gene expressions in a single reaction. By utilising next-generation sequencing technology and automation, HTS^2^ enhances the parallel processing of samples and genes by directly detecting gene expression in cell lysates (Li et al., 2012; Wang et al., 2023). This approach has been successfully employed to discover new drugs for treating prostate cancer (Li et al., 2012), breast cancer lung metastasis (Shao et al., 2019), and combination immunotherapy for triple-negative breast cancer (Wang et al., 2021).

**FIGURE 1:**
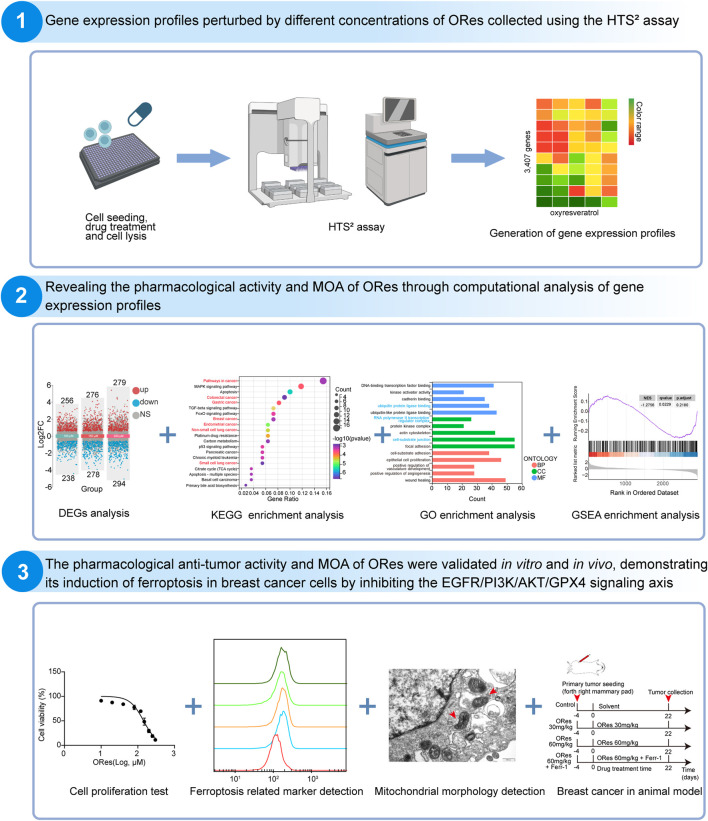
Flowchart of this study. Image generated in BioRender.

Given its promising therapeutic potential, this study aimed to investigate the MOA of ORes in inhibiting breast cancer growth. The workflow for this study is outlined in Figure 1. We generated three ORes-perturbed GEP datasets at different concentrations using HTS^2^. For MOA prediction, we used Gene Ontology (GO) enrichment analysis, KEGG pathway analysis, and Gene Set Enrichment Analysis (GSEA), along with gene signature query tools from LINCS L1000 and Drug Gene DataBase (DGDB). To validate ORes’s effects on ferroptosis in breast cancer cells, we performed CCK-8 assays, colony formation assays, and flow cytometry to assess cell viability and growth. We also measured ferroptosis-related markers (Fe^2+^, reactive oxygen species (ROS), lipid peroxidation) via flow cytometry. Western blotting analysis was used to assess GPX4 expression and key signaling proteins in the EGFR/PI3K/AKT pathway. Transmission electron microscopy was used to observe mitochondrial changes characteristic of ferroptosis. *In vivo*, we established a breast cancer model to assess the effects of ORes on tumor growth by monitoring tumor volume, weight, and histopathological changes. Additionally, immunohistochemistry (IHC) was used to evaluate GPX4 expression in breast cancer tissues. Overall, our findings demonstrate that ORes is a novel ferroptosis inducer that exerts anti-breast cancer effects by inhibiting the activation of the EGFR/PI3K/AKT signaling pathway.

## 2 Materials and methods

### 2.1 Cell culture

MDA-MB-231, 4T1, and BT-549 cells were obtained from ATCC (United States). MDA-MB-231 and 4T1 cells were cultured in RPMI 1640 medium (Gibco, C11875500BT, United States), while BT-549 cells were cultured in DMEM medium (Gibco, C11995500BT, United States). Both media were supplemented with 10% fetal calf serum (ExCell, FSP500, China) and 100 units/mL penicillin-streptomycin. All cells were incubated at 37°C in a humidified atmosphere with 5% CO_2_.

### 2.2 Cell counting kit-8 (CCK-8) assay

The impact of ORes on the cell viability of breast cancer cells was assessed using the CCK-8 assay (Bioground, BG0025, China). Approximately 3,000 cells were seeded into 96-well plates and treated with either DMSO (vehicle control) or ORes (Chengdu Pusi, China) at the specified concentrations. After treatment, the cells were incubated with CCK-8 solution for 2 h at 37°C. Absorbance was then measured at 450 nm using a Varioskan^®^ Flash microplate reader (Thermo, United States).

### 2.3 HTS^2^ assay

To generate the GEP induced by ORes in MDA-MB-231 cells, we conducted the HTS^2^ assay. Approximately 3,500 cells were plated into each well of a 384-well plate and cultured for 24 h. The cells were then treated with various concentrations of ORes for an additional 24 h. Following treatment, the culture medium was discarded, and lysis buffer was added. An HTS^2^ assay was subsequently performed to evaluate the mRNA levels of 3,407 target genes (Supplementary Table 1), following the protocol described in a previous study (Li et al., 2012). Briefly, after treatment, cell lysates were prepared, and mRNA was captured using biotin-oligo-dT and streptavidin magnetic beads. A set of 3,407 gene probes was then added, which annealed to the target mRNA. These probes were ligated using T4 DNA ligase, and the resulting products were eluted and PCR-amplified to introduce the P5 barcode (i5 index), P7 barcode (i7 index), Illumina P5 adapter, and Illumina P7 adapter. The purified and quantified PCR products were then sequenced using next-generation sequencing.

### 2.4 HTS^2^ data processing

HTS^2^ data undergoes several processing steps: trimming, filtering, UMI extraction, alignment, and count calculation. Initially, FASTQ files are trimmed to the first 48 nucleotides using fastx_trimmer from the FASTX-Toolkit. UMIs are then extracted with umi_tools extract from UMI-tools. The processed reads are aligned to an in-house reference containing 3,407 probes using Bowtie 2 (Langmead and Salzberg, 2012), allowing up to 3 mismatches and reporting only unique alignments. Read groups are identified based on their UMIs using umi_tools group. Finally, UMI and read counts are calculated using in-house shell and Perl scripts. RNA transcript level differences are analyzed using DESeq2, with a *p*-value of < 0.05 and |log2 (fold-change)| ≥ 0.585 set as the criteria for identifying differentially expressed genes (DEGs).

### 2.5 Enrichment analysis

We predicted the pharmacological activity of ORes by performing GO enrichment analysis, KEGG pathway analysis and GSEA. GO enrichment analysis of DEGs was performed using the GO database (http://www.geneontology.org). For KEGG enrichment analysis, the KEGG database (http://www.genome.jp/kegg) was utilized. Additionally, GSEA was conducted using the GSEA software with the KEGG gene set, as previously described (Gao et al., 2022). Pathways with a *p*-value < 0.05 in the hypergeometric test were considered significantly enriched.

### 2.6 Colony formation assay

Colony formation assays were performed to assess the colony-forming ability of cells treated with ORes. Cells were seeded into each well of a 6-well plate at a density of 500 cells per well. After seeding, the cells were treated with ORes and then cultured in a medium supplemented with 10% fetal calf serum for 8 days. At the end of the culture period, the resulting colonies were fixed with 4% paraformaldehyde and stained with crystal violet (Beyotime, C0071L, China).

### 2.7 Flow cytometry assay

To evaluate the effects of ORes treatment on proliferation, Fe^2+^ levels, ROS content, and lipid peroxidation in breast cancer cells, we performed flow cytometry analysis. Cells were seeded in 6-well culture plates at a density of 1 × 10^5^ cells per well and incubated for 24 h. Following this initial incubation, the cells were treated with ORes for an additional 24 h. Cell proliferation rates were determined using the BeyoClick™ EdU-488 Cell Proliferation Detection Kit (Beyotime, C0071L, China). Intracellular ROS levels were quantified using a ROS Detection Kit (Beyotime, S0033, China), and lipid peroxidation was assessed with C11-BODIPY581/591 (Dojindo, L267, Japan). All procedures were performed according to the manufacturers’ instructions. Flow cytometry analysis was conducted using a BD FACSVerse system (BD Biosciences, United States), and data were analyzed with FlowJo software version 10 (FlowJo, United States).

### 2.8 Cellular iron detection

Intracellular Fe^2+^ levels were measured using FerroOrange (Dojindo, F-374, Japan). Cells were seeded in 6-well plates and treated with ORes for 24 h. Following the treatment, FerroOrange in serum-free medium was added, and the cells were incubated for 30 min at 37°C. Fluorescence intensity was then evaluated using a fluorescence microscope (Olympus, Hamburg, Germany) and a flow cytometer (BD Biosciences, United States), according to the manufacturer’s instructions.

### 2.9 Western blot analysis

The impact of ORes on the expression levels of key proteins in the EGFR/PI3K/AKT/GPX4 signalling axis was assessed using Western blot analysis. Cells were lysed in RIPA buffer (Beyotime, P0013K, China) with the addition of a protease inhibitor cocktail (Boster, AR1182, China). Total protein concentrations were determined using a BCA assay kit (Boster, AR1189A, China). Proteins were then separated by SDS-PAGE and transferred onto PVDF membranes (Millipore, United States). The membranes were incubated overnight at 4°C with primary antibodies against GPX4 (Cell Signaling Technology, 59735, 1:1,000), phosphorylated EGFR (ZenBio, R26283, 1:1,000), EGFR (Selleck, A5858, 1:1,000), phosphorylated PI3K (ZenBio, 310164, 1:1,000), PI3K (ZenBio, 200900, 1:1,000), phosphorylated AKT (Cell Signaling Technology, 13038, 1:1,000), and AKT (Cell Signaling Technology, 4691, 1:1,000). Following this, a goat anti-rabbit HRP-conjugated secondary antibody was applied for 1 h at 37°C. Immunodetection was performed using an ECL kit (Thermo Scientific, United States), and the signals were visualized using a chemiluminescent imaging system (SAGECREATION, Beijing, China) or Synoptics (Cambridge, United Kingdom).

### 2.10 Mitochondrial morphology

To observe the morphological changes associated with mitochondrial ferroptosis in breast cancer cells, the following transmission electron microscopy (TEM) procedure was employed: cell fixation, post-fixation, dehydration, embedding, sectioning, staining, and TEM examination. Images were captured using a transmission electron microscope (JEOL, JEM-1400FLASH, Japan). For specific experimental details, please refer to the methods described in previous research (Zeng et al., 2016).

### 2.11 Animal studies

To evaluate the inhibitory effects of ORes on *in vivo* breast cancer growth, we conducted animal studies. All animal handling procedures were approved by the Animal Welfare Committee of Chengdu University of Traditional Chinese Medicine (approval number 2023037) and strictly adhered to institutional animal care guidelines. Female Balb/c mice (5 weeks old, 14–16 g) were obtained from HFK Biotechnology Co., Ltd. (China) and housed in a pathogen-free facility with a 12-hour light/dark cycle and unrestricted access to food and water. An orthotopic mammary tumor model was established by injecting 5 × 10^4^ 4T1 cells into the fourth pair of mammary fat pads. The mice were randomly assigned to four groups (n = 8 per group): a control group (castor oil: ethanol: saline = 0.5:0.5:9), an ORes treatment group (30 mg/kg, once daily, intraperitoneally), a second ORes treatment group (60 mg/kg, once daily, intraperitoneally), and a combination treatment group receiving ORes (60 mg/kg, once daily, intraperitoneally) and Ferr-1 (10 mg/kg, once daily, intraperitoneally). After 22 days of treatment, the mice were sacrificed, and the tumors were excised, photographed, and weighed. Tumor tissues were then fixed in 4% paraformaldehyde for further analysis.

### 2.12 Hematoxylin-eosin (HE) staining

The purpose of the HE staining experiment in this study was to evaluate the histopathological changes in breast cancer following ORes treatment. Tumor tissues were fixed in 4% paraformaldehyde for 48 h, dehydrated through a graded alcohol series, and embedded in paraffin. Approximately 6-μm sections were then dewaxed in xylene, rehydrated, and stained with hematoxylin. Differentiation was performed using 1% hydrochloric acid alcohol, followed by counterstaining with 5% eosin. The sections were further dehydrated through graded alcohols, cleared in xylene, and sealed with neutral gum. Detailed histological evaluation was conducted using a NanoZoomer S-60 Digital Slide Scanner (Hamamatsu, Japan).

### 2.13 Immunohistochemistry

To assess the expression levels of GPX4 protein in breast cancer tissues, we performed immunohistochemistry. Immunohistochemistry was performed using a Key-GEN immunohistochemistry kit (KGOS60, China). Paraffin-embedded breast cancer sections were prepared, dewaxed, and subjected to antigen retrieval with 3% citric acid, followed by blocking with goat serum. The sections were then incubated with a primary antibody against GPX4 (Zenbio, 381958, 1:100) for 2 h at 37°C, followed by a 30-min incubation with goat anti-rabbit IgG (Beyotime, A0277, 1:200) at 37°C. Color development was achieved using DAB reagent for 40 s. The sections were then counterstained with hematoxylin, dehydrated, sealed with neutral gum, and imaged using the NanoZoomer S-60 Digital Slide Scanner.

### 2.14 DAB-enhanced Prussian blue staining

DAB-enhanced Prussian Blue staining was employed to detect iron deposition in breast cancer sections. Briefly, the sections were washed with PBS, permeabilized with 0.5% PBS/Triton, and incubated for 1 h in a solution of 4% potassium ferrocyanide and 4% hydrochloric acid. The sections were then treated with DAB and H_2_O_2_ for 10 min, followed by another PBS wash. Images were captured using the NanoZoomer S-60 Digital Slide Scanner.

### 2.15 LINCS L1000 and DGDB query

To predict the MOA of ORes, we utilized LINCS L1000 (https://portals.broadinstitute.org/cmap/) and DGDB (https://www.iomicscloud.com/) to identify compounds with similar pharmacological activities and MOA to ORes. A set of 233 DEGs was used as the gene signature for ORes and input into the “Query” module of LINCS L1000 and the “Gene Signature Query” of DGDB. We then analyzed the targets of the top ten compounds from the query results of both platforms.

### 2.16 siRNA transfection

The purpose of the EGFR siRNA transfection experiment was to investigate the role of EGFR inhibition in regulating the effects of ORes on breast cancer cell. Reverse siRNA transfection was performed using SMARTpool siRNA targeting EGFR (Dharmacon, Lafayette, CO, United States). The sequences of the siRNAs used were as follows: 5′-CAA​AGU​GUG​UAA​CGG​AAU​A-3′, 5′-CCA​UAA​AUG​CUA​CGA​AUA​U-3′, 5′-GUA​ACA​AGC​UCA​CGC​AGU​U-3′, and 5′-CAG​AGG​AUG​UUC​AAU​AAC​U-3′. DharmaFECT 1 transfection reagent (Dharmacon, Lafayette, CO, United States) was diluted in Opti-MEM and added to the siRNA wells. The siRNA-reagent complex was allowed to form while MDA-MB-231 cells were trypsinized and prepared for plating. Subsequently, the cells (1,500 cells per well in a 384-well plate) were seeded into the wells containing the EGFR siRNA complex. Cells were incubated for 72 h before experimental assays were conducted.

### 2.17 Survival analysis

We analyzed the association of *EGFR*, *PTEN*, *INPP4B*, *PIK3CA*, *AKT3*, and *GPX4* expression with overall survival (OS) in breast cancer patients using RNA-seq datasets from the KM plotter breast cancer sample database (https://kmplot.com/).

### 2.18 Statistical analysis

Data are expressed as mean ± SD. The two-tailed unpaired Student’s *t*-test was used to compare differences between two groups. For comparisons among multiple groups, one-way ANOVA was applied. Data analysis was performed using GraphPad Prism 9.0 (GraphPad Software, Inc.). Statistical significance was indicated as *p < 0.05, **p < 0.01, and ***p < 0.001, ****p < 0.0001.

## 3 Results

### 3.1 ORes inhibits the growth of breast cancer cells *in vitro*

**FIGURE 2:**
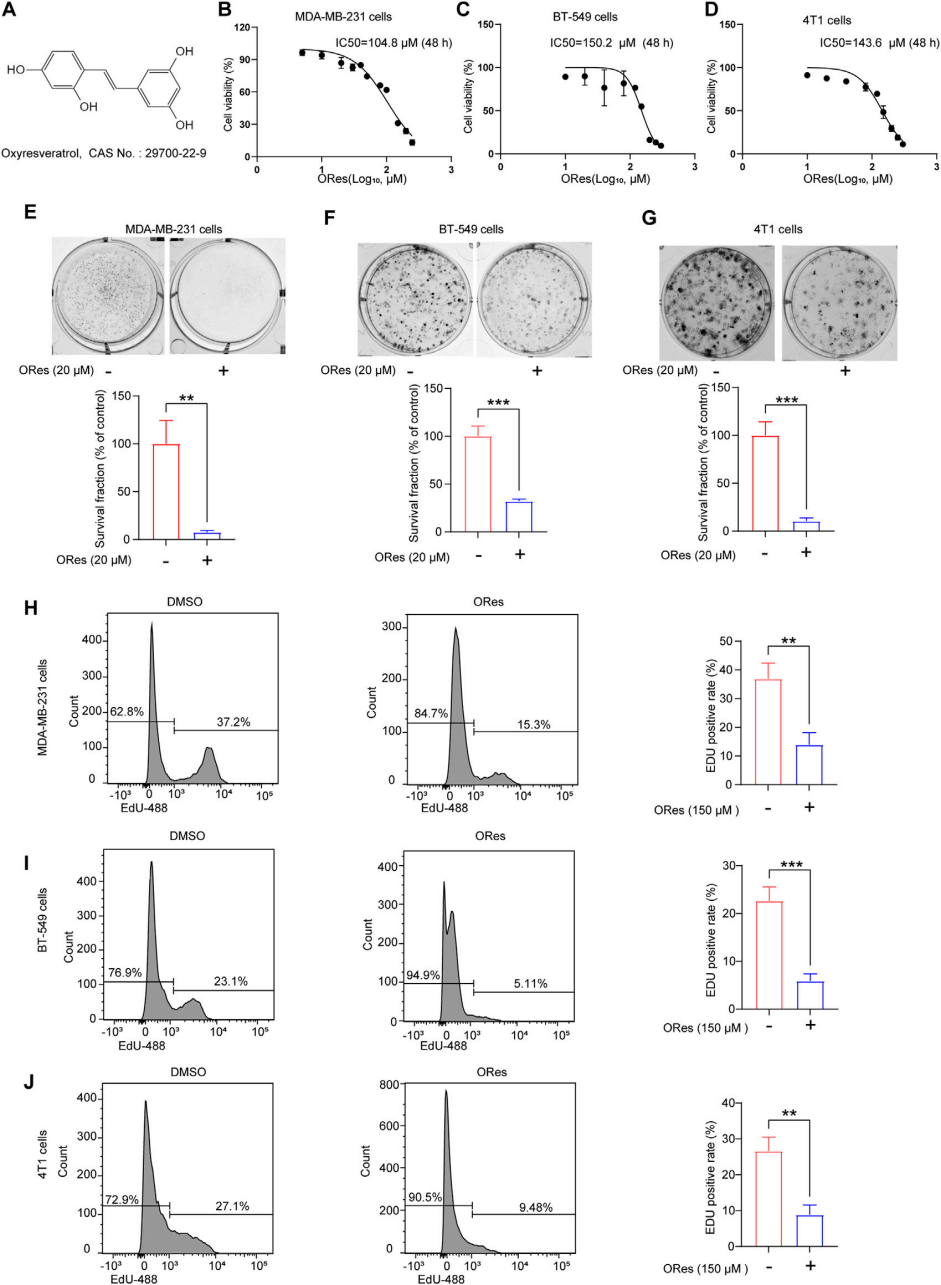
ORes inhibits the growth of breast cancer cells *in vitro*. **(A)** Molecular structure of ORes. **(B)** IC_50_ of ORes in MDA-MB-231 cells. **(C)** IC_50_ of ORes in BT-549 cells. **(D)** IC_50_ of ORes in 4T1 cells. **(E)** Representative images (upper panel) and quantitative analysis (lower panel) of MDA-MB-231 cells clone formation after ORes treatment. **(F)** Representative images (upper panel) and quantitative analysis (lower panel) of BT-549 cells clone formation after ORes treatment. **(G)** Representative images (upper panel) and quantitative analysis (lower panel) of 4T1 cell clone formation after ORes treatment. **(H–J)** Left and middle panel: positive rate of EdU-488 in MDA-MB-231 **(H)**, BT-549 **(I)**, and 4T1 **(J)** cells detected by flow cytometry. Right panel: quantitative analysis of the left panel. Experiments were performed in triplicate, and data are presented as mean ± SD. **p < 0.01; ***p < 0.001.

We first confirmed the inhibitory effects of ORes (molecular structure presented in Figure 2A) on breast cancer cell growth *in vitro* and explored the effective concentration range for subsequent experiments. Three breast cancer cell lines (MDA-MB-231, BT-549, and 4T1) were treated with various concentrations of ORes. The results indicated a concentration-dependent decrease in cell viability, with IC_50_ values of 104.8 μM, 150.2 μM, and 143.6 μM, respectively (Figures 2B–D). To further assess the effect of ORes on cell proliferation, a colony formation assay was performed. This assay revealed significant (*p* < 0.05) inhibition of colony formation in all three breast cancer cell lines (Figures 2E–G). In addition, flow cytometry was used to assess the proliferation of ORes in breast cancer cells. The results indicated a significant (*p* < 0.05) decrease in the proportion of proliferating cells, and this was consistent with the CCK-8 and colony formation assay results (Figures 2H–J). Collectively, these findings demonstrate that ORes effectively inhibited the growth of breast cancer cells *in vitro*.

### 3.2 Enrichment of PI3K/AKT and ferroptosis signalling pathways following ORes treatment

**FIGURE 3:**
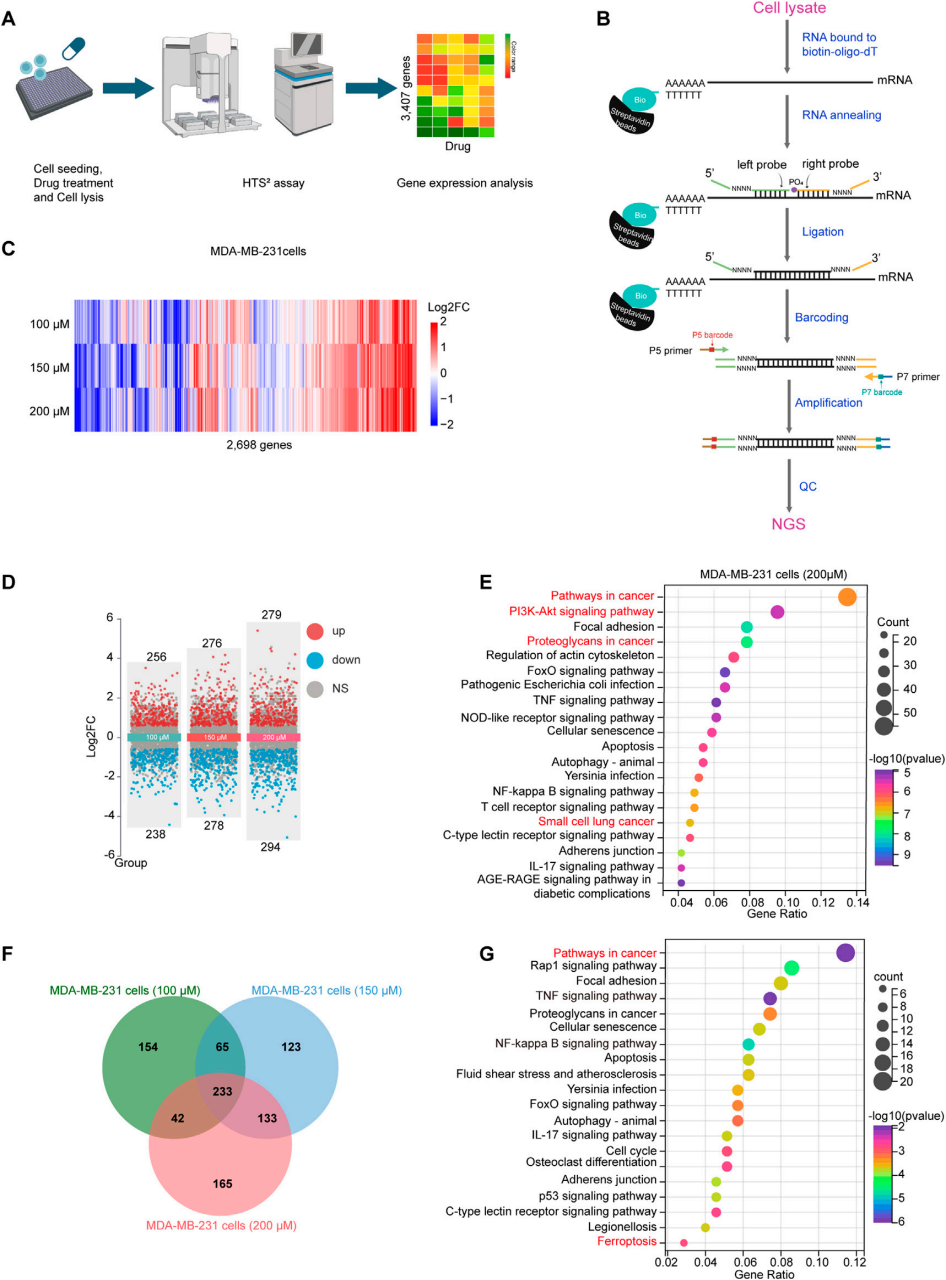
Enrichment of PI3K/AKT and ferroptosis signalling pathway following ORes treatment. **(A)** Flow diagram illustrating the HTS^2^ assay. This image was created using BioRender. **(B)** Schematic representation of the HTS^2^ assay. This image was created using BioRender. **(C)** Heatmap depicting the gene expression patterns in MDA-MB-231 cells following perturbation with different concentrations of ORes. Out of 3,407 genes analysed, 2,698 genes were expressed in MDA-MB-231 cells. **(D)** Volcano plot indicating DEGs in MDA-MB-231 cells. Differential gene screening criteria: |FoldChange|>1.5 and *p-*value < 0.05. **(E)** Bar plots displaying KEGG pathway enrichment analysis of DEGs in MDA-MB-231 cells treated with 200 μM ORes. **(F)** Venn diagram illustrating the DEGs among three treatment groups in MDA-MB-231 cells, with 233 overlapping genes. **(G)** Bar plots displaying KEGG enrichment analysis of the 233 overlapping DEGs.

To investigate the MOA of the ORes, we conducted an HTS^2^ assay. The experimental workflow and biochemical principles underlying the HTS^2^ assay that utilises targeted capture sequencing are depicted in Figures 3A, B. Given the IC_50_ of ORes in breast cancer cells (ranging from 100 μM to 200 μM), we generated GEPs for MDA-MB-231 cells treated with ORes at concentrations of 100 μM, 150 μM, and 200 μM (Figure 3C). Of the 3,407 genes analysed, 2,698 were expressed in MDA-MB-231 cells.

First, we performed differential expression analysis for these GEPs and identified DEGs across various concentration groups. The number of DEGs progressively increased with higher concentrations (Figure 3D). KEGG pathway enrichment analysis revealed significant enrichment in tumour-related pathways and the PI3K/AKT signalling pathway at all three concentrations (Figure 3E; Supplementary Figures 1A, B). Furthermore, GSEA suggested that ORes inhibited these tumour-related signalling pathways (Supplementary Figures 1C–G). GO enrichment analysis also indicated that ORes significantly modulated the pathways involved in tumourigenesis and tumour progression (Supplementary Figures 2A–C). These findings corroborate our *in vitro* observations of ORes-mediated growth inhibition of breast cancer cells.

In total, 233 overlapping DEGs were identified across the three datasets (Figure 3F). These 233 DEGs exhibited consistent expression patterns across all three profiles (Supplementary Figure 2D), with Pearson correlation coefficients of greater than 0.9 between each pair (Supplementary Figure 2E). This subset of 233 genes constituted the gene signature of ORes that was significantly enriched in pathways related to cancer and ferroptosis (Figure 3G). Given that ferroptosis has emerged as a critical area of research, particularly due to its potential as a therapeutic strategy against tumors, including those resistant to traditional treatments (Gao et al., 2019; He et al., 2023), we hypothesize that the MOA through which ORes exerts its anti-breast cancer activity is closely related to the induction of ferroptosis and the modulation of the PI3K/AKT signalling pathway. We now aim to validate this hypothesis through further investigation.

### 3.3 ORes increases ferrous iron levels in breast cancer cells

**FIGURE 4:**
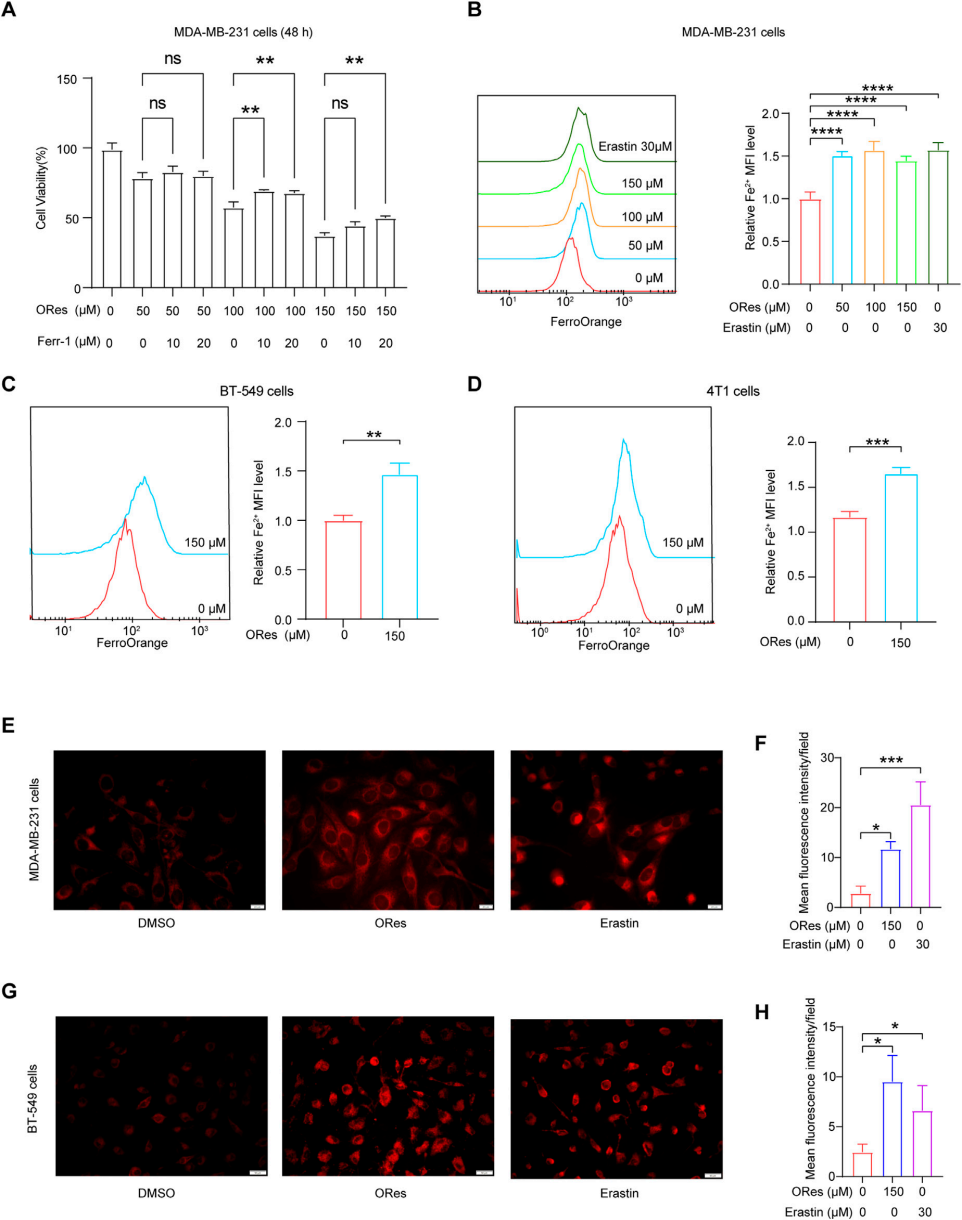
ORes increases ferrous iron levels in breast cancer cells. **(A)** Cell proliferation as measured by the CCK8 assay. **(B–D)** Left panel: determination of intracellular ferrous iron levels using FerroOrange after ORes treatment in MDA-MB-231 **(B)**, BT-549 **(C)**, and 4T1 cells **(D)**. Right panel: quantitative analysis of the left panel. **(E)** Intracellular ferrous ions in MDA-MB-231 cells as visualized by fluorescence microscopy. Scale bar, 20 μm. **(F)** Quantitative analysis of panel **(E)**. **(G)** Intracellular ferrous ions in BT-549 cells as visualized by fluorescence microscopy. Scale bar, 50 μm. **(H)** Quantitative analysis of panel **(G)**. Experiments were performed in triplicate, and data are presented as mean ± SD. *p < 0.05, **p < 0.01, and ***p < 0.001, ****p < 0.0001. ns, no significance.

To further explore the link between ORes-induced cell death and ferroptosis in breast cancer cells, we performed the CCK-8 assay. The results demonstrated that ORes induced cell death in a concentration-dependent manner (Figure 4A), and this effect was effectively blocked by the ferroptosis inhibitor Ferr-1 (*p* < 0.05), suggesting a ferroptosis-related mechanism. Ferroptosis is a form of regulated cell death characterised by the accumulation of ferrous iron (Yu et al., 2022). To quantify cellular ferrous iron levels, we performed FerroOrange staining followed by flow cytometry analysis. The results revealed a concentration-dependent (*p* < 0.05) increase in the ferrous iron content in MDA-MB-231 (Figure 4B), BT-549 (Figure 4C), and 4T1 (Figure 4D) cells in response to ORes treatment. Similar results were observed for ferrous iron staining, where ORes treatment exacerbated the accumulation of ferrous iron in both MDA-MB-231 cells (*p* < 0.05) (Figures 4E, F) and BT-549 cells (Figures 4G, H). Notably, the ability of ORes to induce ferrous iron accumulation was comparable to that of erastin, a well-known ferroptosis inducer (Nebie et al., 2019). These results provide strong evidence that ORes promotes ferroptosis in breast cancer cells by increasing intracellular ferrous iron levels.

### 3.4 ORes elevates ferroptosis-related ROS levels and lipid peroxidation in breast cancer cells

**FIGURE 5:**
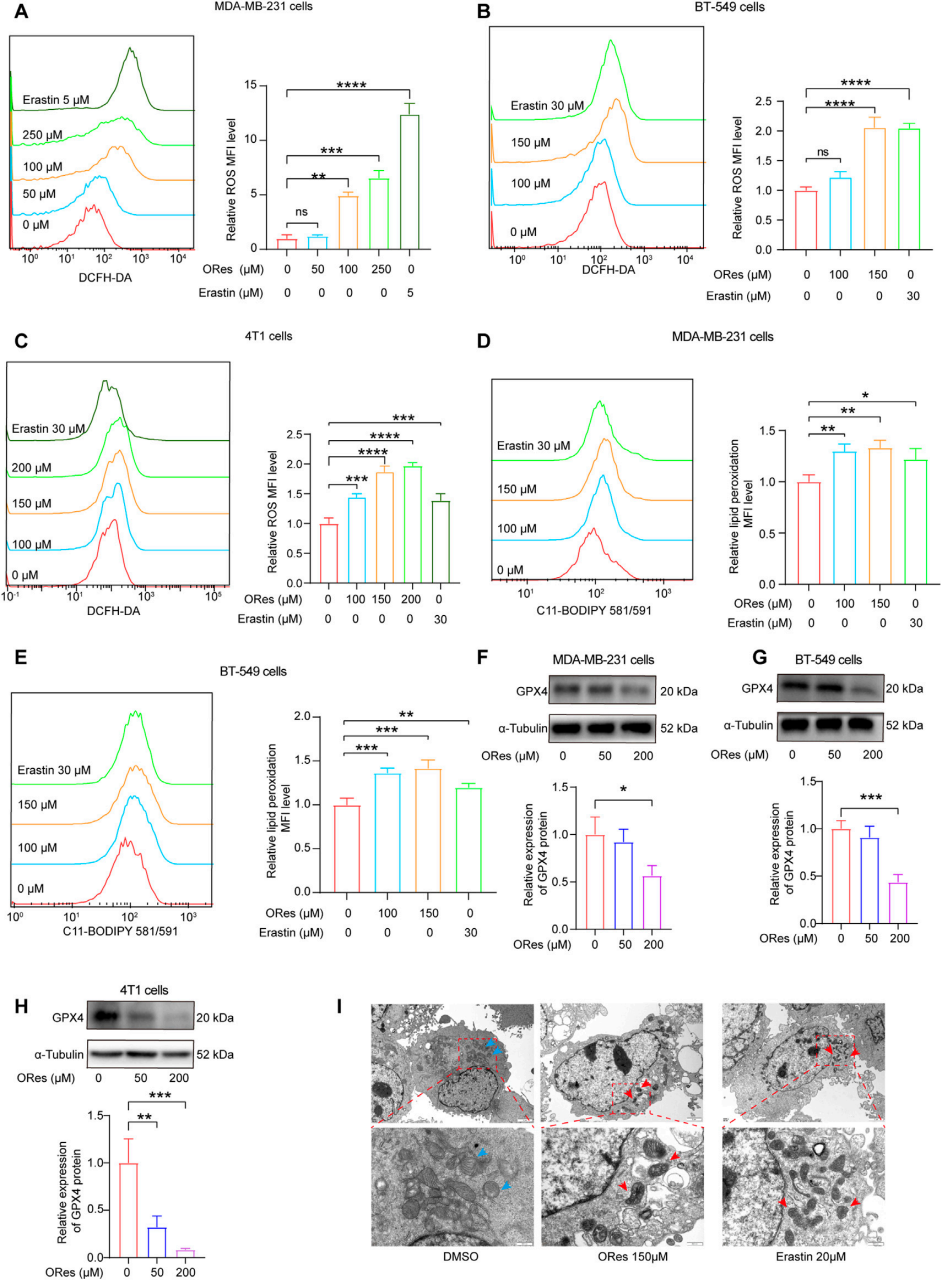
ORes elevates ferroptosis-related ROS levels and lipid peroxidation in breast cancer cells. **(A–C)** Left panel: determination of intracellular ROS levels using DCFH-DA after ORes treatment in MDA-MB-231 **(A)**, BT-549 **(B)**, and 4T1 cells **(C)**. Right panel: quantitative analysis of the left panel. **(D, E)** Left panel: determination of lipid peroxidation levels using the lipid peroxidation probe C11-BODIPY581/591 after ORes treatment in MDA-MB-231 **(D)** and BT-549 **(E)** cells. Right panel: quantitative analysis of the left panel. **(F–H)** Protein expression levels of GPX4 after ORes treatment in MDA-MB-231 **(F)**, BT-549 **(G)**, and 4T1 **(H)** cells. Upper panel: representative Western blot. Lower panel: quantification of western blots. **(I)** Representative photomicrographs of transmission electron microscopy in MDA-MB-231 cells. Blue arrows indicate normal mitochondria, while red arrows indicate abnormal mitochondrial morphology typical of ferroptosis. Upper scale bar = 2 μm, lower scale bar = 500 nm. Experiments were performed in triplicate, and data are presented as mean ± SD. *p < 0.05; **p < 0.01; ***p < 0.001; ****p < 0.0001; NS, no significance. DCFH-DA, 2,7-dichlorofluorescein diacetate. C11-BODIPY581/591, 4,4-difluoro-5-(4-phenyl-1,3-butadienyl)-4-bora-3a,4a-diaza-s-indacene-3-propionic acid.

The accumulation of ferrous iron triggers lipid peroxidation, leading to cellular ferroptosis (Zhang et al., 2022). Additionally, the buildup of ROS is a crucial factor in initiating ferroptosis (Huang et al., 2019). We used a DCFH-DA ROS fluorescent probe to detect intracellular ROS generation and a C11 BODIPY 581/591 fluorescent probe to measure lipid peroxidation using flow cytometry. The results (Figures 5A–C) revealed that ORes induced ROS generation in breast cancer cells in a concentration-dependent manner (*p* < 0.05). Furthermore, ORes significantly (*p* < 0.05) increased lipid peroxidation in both MDA-MB-231 (Figure 5D) and BT-549 (Figure 5E) cells in a concentration-dependent manner.

GPX4, a key regulator of ferroptosis, plays a crucial role as a biomarker of this process (Fei et al., 2020). We investigated intracellular GPX4 levels by Western blotting and observed that ORes treatment decreased GPX4 expression compared to that in the control group, indicating inactivation of the GPX4 signalling pathway (Figures 5F–H). Additionally, transmission electron microscopy revealed distinct mitochondrial alterations in ORes-treated breast cancer cells, including fractured or absent cristae and shrunken mitochondria with increased membrane densities (Figure 5I). In summary, ORes induces ferroptosis in breast cancer cells through inactivation of GPX4, accumulation of ROS, increased iron levels, and enhanced lipid peroxidation.

### 3.5 ORes inhibits breast cancer growth through inducing ferroptosis *in vivo*

**FIGURE 6:**
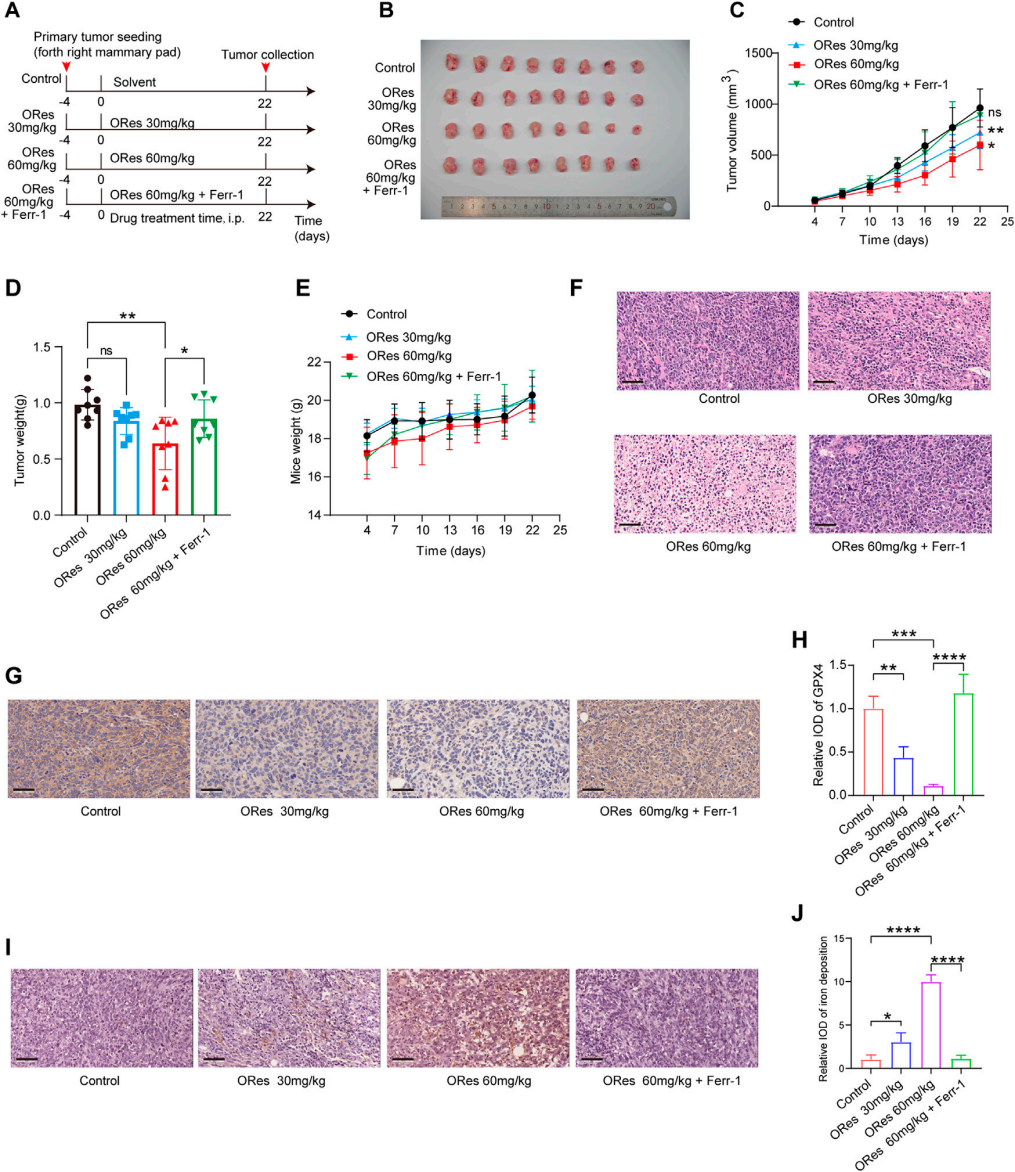
ORes inhibits breast cancer growth *in vivo* through inducing ferroptosis. **(A)** Schematic of the experimental design in mice, with Ferr-1 treatment dosage set at 10 mg/kg. **(B)** Photographs of harvested tumours (n = 8 per group). **(C)** Tumour growth curves (n = 8 per group). **(D)** Weights of the harvested tumours (n = 8 per group). **(E)** Body weight changes of mice in each group during treatment (n = 8 per group). **(F)** Representative HE staining images. **(G)** Representative IHC staining indicating GPX4 expression in tumours. Scale bar, 50 μm. **(H)** Quantification of IHC staining (n = 3 per group). **(I)** DAB-enhanced Prussian blue staining detecting iron deposition in tumours. Scale bar, 50 μm. **(J)** Quantification of DAB-enhanced Prussian blue staining (n = 3 per group). All data are presented as mean ± SD. *p < 0.05; **p < 0.01; ***p < 0.001; ****p < 0.0001. ns, not significant.

After confirming that ORes induces ferroptosis to inhibit breast cancer cell growth *in vitro*, we validated these findings *in vivo* using a breast cancer animal model. A schematic representation of animal experiments is presented in Figure 6A. Both low-dose (30 mg/kg) and high-dose (60 mg/kg) ORes treatments significantly (*p* < 0.05) inhibited tumour volume compared to the control group (Figures 6B, C). Notably, only the high-dose ORes group exhibited a statistically significant (*p* < 0.05) reduction in tumour weight (Figure 6D). Additionally, analysis of the body weight change curve indicated that the ORes had no significant effect on the mice’s body weight (Figure 6E).

Microscopic analysis of HE-stained tumour sections revealed that cells in the control group were tightly packed, whereas ORes-treated tumours displayed loosely arranged cells (Figure 6F). Importantly, the inhibitory effect of ORes on tumour growth was reversed by Ferr-1 treatment (*p* < 0.05) (Figures 6B–D, F). Next, we examined markers of ferroptosis. Immunohistochemical staining for the ferroptosis marker GPX4 in breast tumour tissues indicated a significant (*p* < 0.05) downregulation of GPX4 in the ORes treatment group (Figures 6G, H). DAB-enhanced Prussian blue staining indicated significant (*p* < 0.05) iron deposition in breast cancer tissues after ORes treatment (Figures 6I, J). Notably, the downregulation of GPX4 expression and induction of iron deposition by ORes were reversed by Ferr-1 treatment. These results demonstrate that ORes induces ferroptosis by inactivating GPX4, thereby inhibiting tumour growth *in vivo*.

### 3.6 ORes induces ferroptosis in breast cancer cells via EGFR/PI3K/AKT/GPX4 signalling axis

**FIGURE 7:**
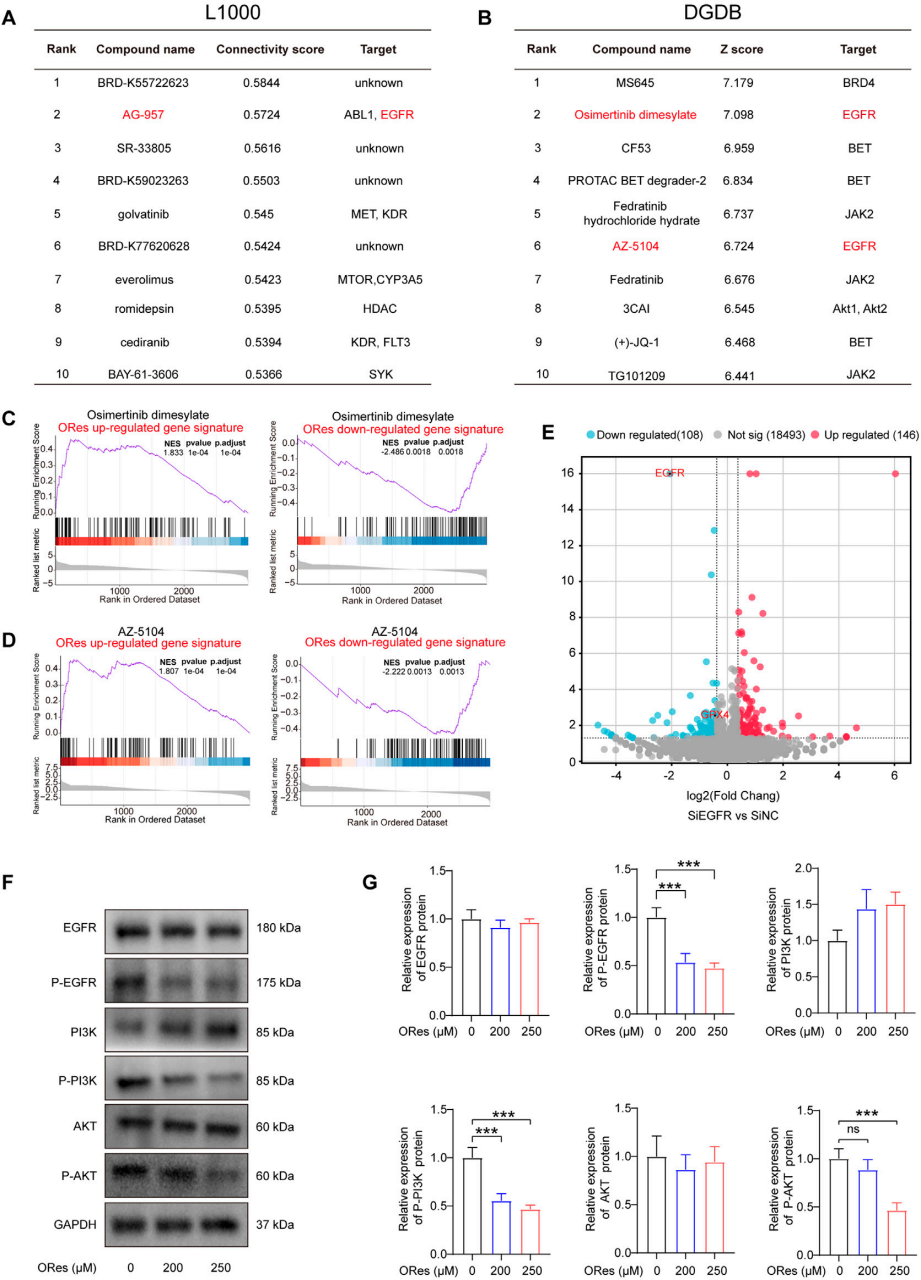
ORes induces ferroptosis in breast cancer cells via the EGFR/PI3K/AKT/GPX4 signalling axis. **(A)** The table indicates the top 10 connectivity scores between the input gene signature and the gene signatures of compounds in the LINCS L1000 touchstone dataset. **(B)** The table displays the top 10 scores between the input gene signature and the gene signatures of compounds in the DGDB dataset. **(C)** GSEA of HTS^2^ results of Osimertinib dimesylate–treated MDA-MB-231 cells. **(D)** GSEA of HTS^2^ results of AZ-5104–treated MDA-MB-231 cells. **(E)** Volcano plot of the DEGs in EGFR knockdown MDA-MB-231 cells, with red and blue dots indicating upregulated and downregulated genes, respectively. Differential gene screening criteria: |FoldChange|>1.3 and *p-*value < 0.05. **(F)** Representative Western blot results. **(G)** Quantification of western blots. Experiments were performed in triplicate, and data are presented as mean ± SD. ***p < 0.001; ns, no significance.

To elucidate the mechanism by which ORes induces ferroptosis and exerts their anti-breast cancer effects, we analysed LINCS L1000 and DGDB datasets. The results revealed that AG-957, Osimertinib dimesylate, and AZ-5104 were among the top ten compounds with GEPs similar to those induced by ORes in both databases (Figures 7A, B). Moreover, we observed that the GEPs of Osimertinib dimesylate and AZ-5104 in the DGDB were positively enriched in the ORes gene signature (Figures 7C, D). As all of these compounds inhibit EGFR protein function (Sun et al., 2001; Cross et al., 2014), we hypothesised that ORes, similar to these three compounds, facilitates anti-breast cancer activity by inhibiting EGFR.

KEGG enrichment analysis revealed that ORes significantly enriched the downstream pathways of EGFR, particularly the PI3K/AKT signalling pathway (Figure 3E; Supplementary Figures 1A, B). The PI3K/AKT pathway plays a critical role in regulating essential cellular processes in various cancers (Yang et al., 2019), and evidence suggests that inhibiting this pathway enhances cancer cell sensitivity to ferroptosis (Yi et al., 2020). Moreover, the PI3K/AKT pathway mediates GPX4 function (Shao et al., 2022) and regulates ferroptosis in tumour cells (Li J. et al., 2023).

Notably, EGFR knockdown in MDA-MB-231 cells reduced GPX4 expression (Figure 7E), indicating that EGFR inhibition lowers GPX4 levels in breast cancer cells. Additionally, our prognostic analysis of genes encoding proteins in the EGFR/PI3K/AKT signalling pathway and GPX4 in breast cancer revealed that reduced activation of the EGFR/PI3K/AKT pathway and decreased GPX4 expression were associated with better patient outcomes (Supplementary Figure 2F). To further validate the regulation of the EGFR/PI3K/AKT/GPX4 signalling axis by ORes in breast cancer cells, we performed Western blot analysis to assess the expression levels of key proteins in this pathway. Our results indicated that ORes exerted a dose-dependent inhibitory effect on the phosphorylation of EGFR, PI3K, and AKT (*p* < 0.05), whereas the total protein levels of EGFR, PI3K, and AKT remained unchanged (Figures 7F, G). Collectively, these findings suggest that ORes induces ferroptosis in breast cancer cells by inhibiting the EGFR/PI3K/AKT/GPX4 signalling axis.

## 4 Discussion

Natural products and their derivatives have long been valuable in drug discovery and therapeutic development (Al-Samydai et al., 2021). ORes, a compound isolated from *Artocarpus lakoocha Roxb* (Choi et al., 2018; Dhakar et al., 2019), possesses significant anti-breast cancer properties (Sunilkumar et al., 2020; Passos et al., 2024). However, the precise MOA of ORes against breast cancer has yet to be fully elucidated. In this study, we investigated the anti-breast cancer pharmacological activity and MOA of ORes by analysing GEPs. Our GEPs analysis revealed that ORes significantly inhibited tumour-related pathways and was enriched in the ferroptosis and PI3K/AKT signalling pathways. Subsequent experiments confirmed that ORes induced ferroptosis by inhibiting GPX4 activity and increasing ROS, ferrous ions, and lipid peroxidation levels in breast cancer cells, thereby inhibiting their growth both *in vivo* and *in vitro*. Furthermore, we discovered that ORes may induce ferroptosis via the EGFR/PI3K/AKT/GPX4 signalling axis.

Current research investigating the pharmacological activity and MOA of ORes often relies on speculative experimental hypotheses (Liu et al., 2018; Tran et al., 2023). However, this approach that is based on preliminary data or limited observations can yield unreliable results, carries a high risk of failure, and consumes significant time and resources. As a result, studies examining the anti-breast cancer activity and MOA of ORes have primarily been limited to *in vitro* studies with few validations *in vivo*. In contrast, analysing GEPs offers a data-driven approach that systematically reveals the complexity and dynamic changes in biological systems. This method has been widely used to identify the pharmacological activities and MOA of various compounds (Subramanian et al., 2017; Tian et al., 2023). To elucidate the MOA of the ORes more clearly, we leveraged the advantages of GEPs in this study. Using HTS^2^, we collected GEPs from MDA-MB-231 cells treated with three different concentrations of ORes to gain a deeper understanding of their anti-breast cancer activity.

We performed multi-scale biological characteristic analyses on the ORes-perturbed GEPs, including differential gene expression analysis, KEGG pathway analysis, GO analysis, and target prediction. Compared to analysing a single concentration, multiscale analysis of GEPs induced by different concentrations provides a comprehensive identification of dose-response relationships and effective dosages (Wang et al., 2016). We observed that higher concentrations of ORes in MDA-MB-231 cells led to increased cell perturbation and a greater number of DEGs. Additionally, within the effective dose range, ORes induced similar gene expression patterns, significantly inhibiting a series of tumour-related pathways, indicating that its antitumour activity was evident within this concentration range. These findings are consistent with our *in vitro* and *in vivo* results, in which the ORes inhibited the growth of breast cancer cells, demonstrating the feasibility of using GEP analysis to study the pharmacological activity and MOA of ORes. Furthermore, MOA predictions based on GEPs indicated that ORes treatment enriched pathways such as the PI3K/AKT signalling pathway and ferroptosis, providing valuable insights for our subsequent exploration of ORes’s MOA.

Ferroptosis is a regulated cell death involving lipid peroxidation and iron-dependent oxidative damage, leading to membrane disruption and cell death (Shi et al., 2022). This process is regulated by key factors such as GPX4, iron metabolism, and lipid peroxidation pathways (Kotschi et al., 2022). Recently, ferroptosis has garnered significant attention as a cancer therapy, particularly in breast cancer (Mou et al., 2019). Traditional therapies, such as surgery, chemotherapy, and radiation, possess limitations, including resistance and significant side effects. The discovery of ferroptosis as a novel cell death pathway presents new therapeutic opportunities (Verma et al., 2020), particularly in the search for natural products that induce ferroptosis in breast cancer cells. In this study, we identified ORes as novel inducers of ferroptosis. ORes significantly inhibited breast cancer growth both *in vitro* and *in vivo*, and the ferroptosis inhibitor Ferr-1 attenuated its inhibitory effects. Furthermore, ORes treatment led to elevated levels of ROS, ferrous ions, and lipid peroxidation in breast cancer cells, along with characteristic ferroptosis-associated mitochondrial morphological changes. These findings provide strong experimental and theoretical support for the potential clinical application of ORes in breast cancer treatment.

GPX4 is a key enzyme in the glutathione peroxidase family that specifically reduces lipid hydroperoxides to their corresponding alcohols, thereby preventing the accumulation of toxic lipid peroxides (Ursini and Maiorino, 2020). When GPX4 activity is compromised, either through genetic knockdown or pharmacological inhibition, cells become susceptible to ferroptosis due to unchecked lipid peroxide accumulation (Xu et al., 2022). This vulnerability is further exacerbated by the presence of iron that facilitates ROS generation via the Fenton reaction, amplifying lipid peroxidation, and ultimately leading to cell death (Sui et al., 2018). Notably, GPX4 inhibition can selectively induce ferroptosis in cancer cells while sparing normal cells that exhibit lower basal lipid peroxidation and stronger antioxidant defences (Huang et al., 2022). For example, RSL3, a known GPX4 inhibitor, effectively induces ferroptosis in tumour cells by directly binding to and inactivating GPX4 (Liu et al., 2021). In our study, we observed a significant decrease in GPX4 protein levels in ORes-treated breast cancer cells and tissues compared to that in controls, suggesting that ORes-induced ferroptosis is mediated by the inhibition of GPX4.

Ferroptosis inducers such as Erastin and Cisplatin have shown promise in cancer therapy but are limited by issues such as poor stability and significant toxicity. In our study, ORes effectively induced ferroptosis in breast cancer cells, exhibiting similar effects to Erastin, including increased ferrous ions, ROS, lipid peroxidation, and mitochondrial alterations. However, ORes demonstrated superior stability and solubility, and unlike Erastin, it is less susceptible to enzymatic degradation (Hu et al., 2014). Additionally, ORes exhibits hepatoprotective properties (Oh et al., 2002; Choi et al., 2016) and is safer than Cisplatin, which is associated with severe toxicities, including nephrotoxicity. Furthermore, ORes regulates the EGFR/PI3K/AKT/GPX4 axis, targeting both ferroptosis and key cancer-related pathways, which distinguishes it from other ferroptosis inducers. For instance, commonly used inducers like RSL3 and Erastin act by directly inhibiting GPX4 and SLC7A11, respectively, but they fail to comprehensively modulate cancer-associated signaling networks (Shin et al., 2018; Kain et al., 2020). These advantages position ORes as a promising ferroptosis inducer with enhanced stability, safety, and therapeutic potential. However, further studies addressing potential off-target effects, inter-patient variability, and clinical translation challenges will be essential to determine the full clinical applicability of ORes.

To better understand the MOA through which ORes induces ferroptosis and inhibits breast cancer cell growth, we conducted further MOA predictions by inputting GEPs into the DGDB and LINCS L1000 databases. Our analysis revealed that ORes shares a similar MOA with AG-957, Osimertinib dimesylate, and AZ-5104 (Sun et al., 2001; Cross et al., 2014), all of which inhibit EGFR function. EGFR is closely related to cell proliferation and development, and its dysregulation leads to the malignant transformation and progression of various cancers. Upon activation, EGFR recruits and activates PI3K, which converts PIP2 to PIP3, leading to AKT activation. This cascade promotes cell survival, proliferation, and growth (Chang et al., 2013; Zhangyuan et al., 2020). Furthermore, studies have showed that the PI3K-AKT pathway mediates GPX4 function (Shao et al., 2022) and regulates ferroptosis in tumour cells (Li J. et al., 2023). In the present study, we determined that EGFR knockdown in breast cancer cells inhibited GPX4 expression. Interestingly, GEPs induced by different concentrations of ORes were significantly enriched in the PI3K-AKT signalling pathway downstream of EGFR, and activation of the EGFR/PI3K/AKT/GPX4 axis was associated with poor prognosis in breast cancer patients. Based on these findings, we hypothesised that ORes exert their effects by inhibiting EGFR, thereby modulating the PI3K-AKT pathway, suppressing GPX4 activity, and inducing ferroptosis in breast cancer cells. This hypothesis was confirmed in our study, which revealed a significant decrease in the levels of phosphorylated EGFR, PI3K, AKT, and GPX4 in ORes-treated breast cancer cells.

## 5 The limitation of the present study

While this study demonstrated that ORes exerted anti-breast cancer activity by inducing ferroptosis through the inhibition of the EGFR/PI3K/AKT/GPX4 signalling axis, it is important to note some limitations. Investigating the impact of EGFR activators on the therapeutic effects of ORes both *in vivo* and *in vitro* would help to further validate the MOA of ORes.

## 6 Conclusion

**FIGURE 8:**
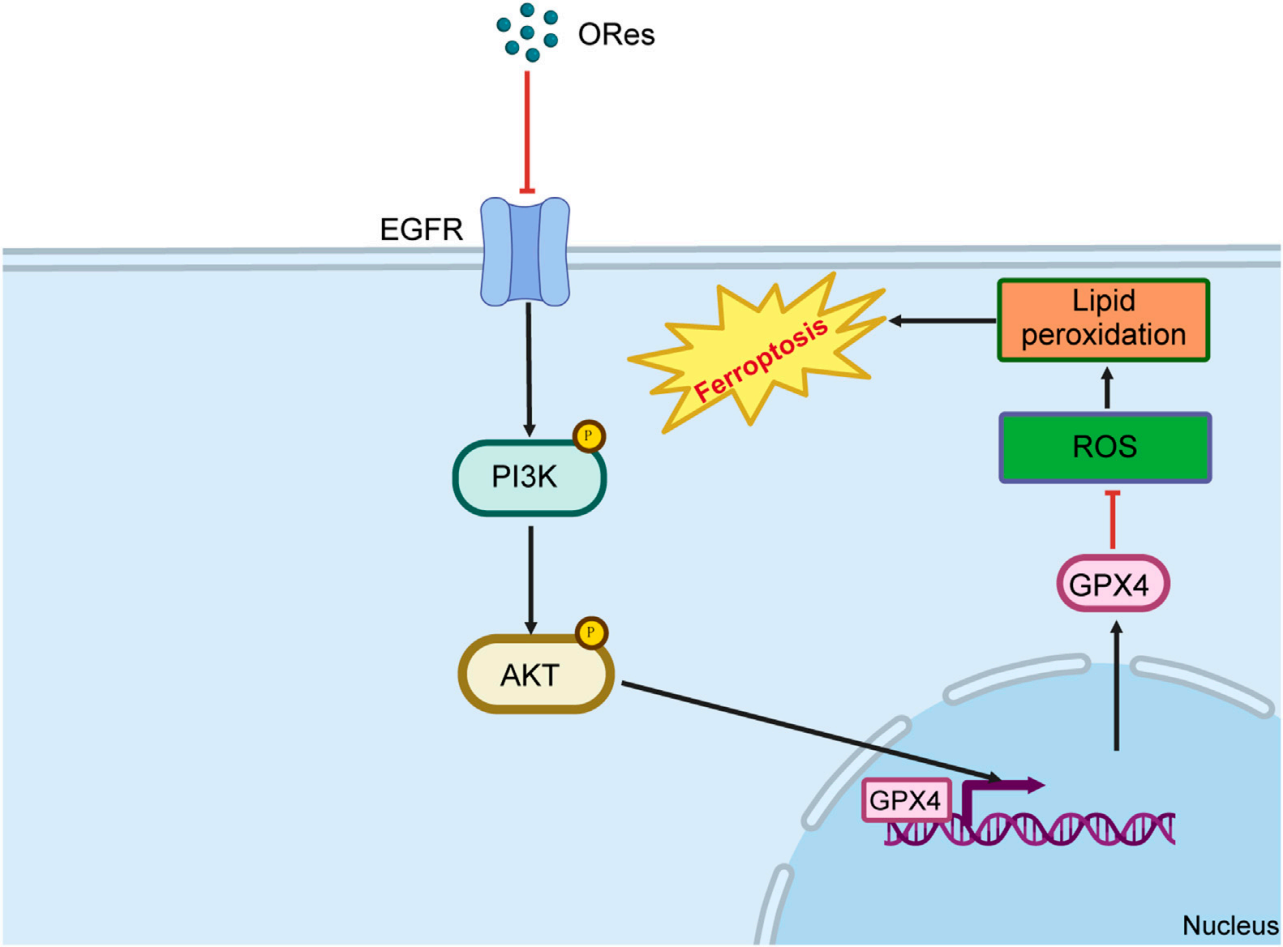
Schematic diagram of the mechanism of ORes inducing ferroptosis. Image generated in BioRender.

In this study, we uncovered that ORes induces ferroptosis in breast cancer cells through the inhibition of the EGFR/PI3K/AKT/GPX4 signalling axis. This inhibition triggers a cascade of events, including elevated cellular ferrous ion levels, ROS accumulation, lipid peroxidation, and mitochondrial damage, all of which converge to drive ferroptosis (Figure 8). These findings position ORes as a promising alternative therapeutic strategy for breast cancer treatment.

## Data Availability

The original contributions presented in the study are publicly available. This data can be found here: https://doi.org/10.6084/m9.figshare.28112525.v1.
