# Supplementary material for: Oxyresveratrol as a novel ferroptosis inducer exhibits anticancer activity against breast cancer via the EGFR/PI3K/AKT/GPX4 signalling axis
Source: Front Pharmacol. 2025 Jan 15;15:1527286. doi: 10.3389/fphar.2024.1527286 (PMC11775479; doi:10.3389/fphar.2024.1527286)
Supplement: Supplementary file 1 [file DataSheet1.pdf]

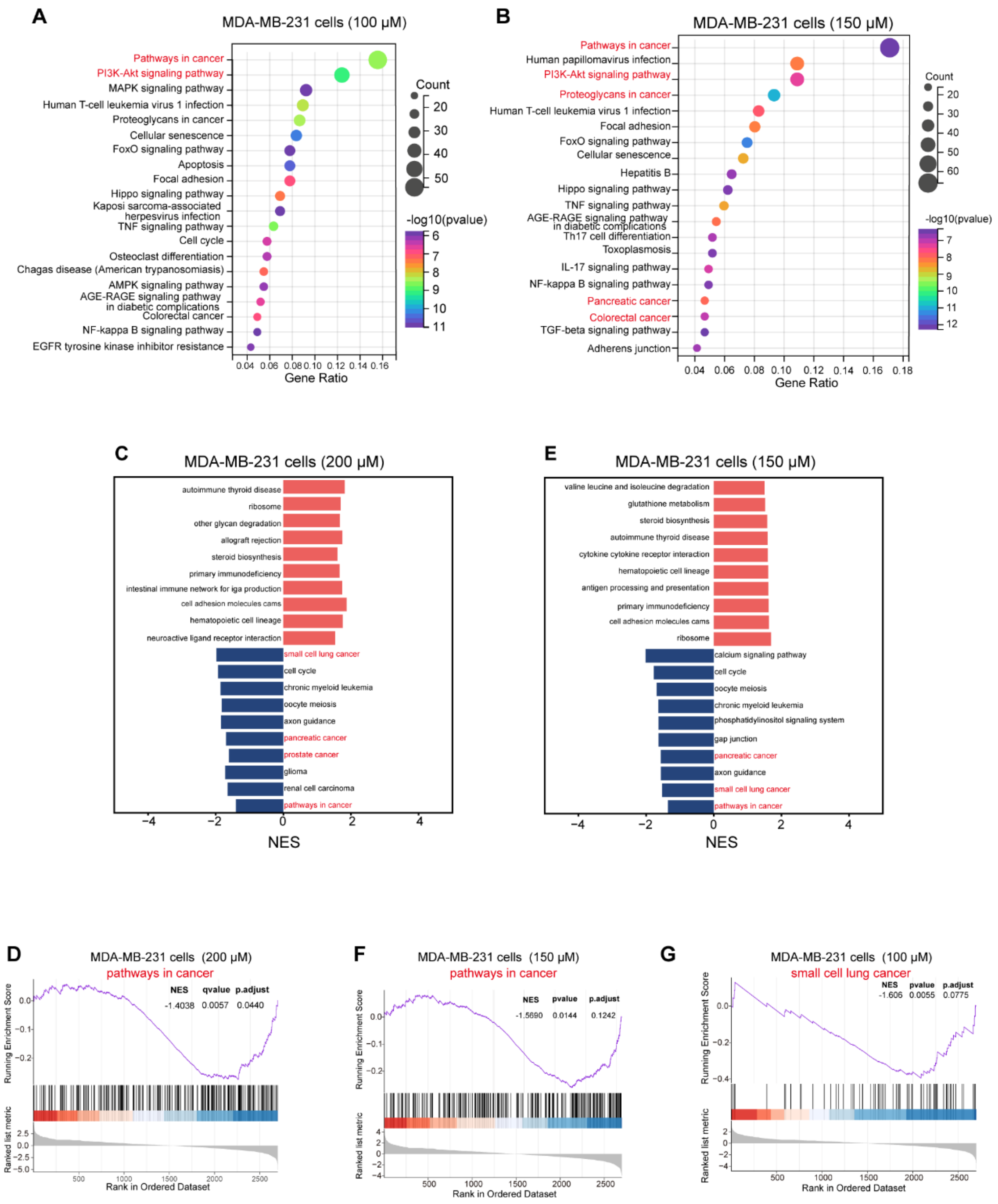

Supplementary Figure 1. ORes inhibits tumour-related pathways. (**A** and **B**) Bar plots displaying KEGG pathway enrichment analysis of DEGs in MDA-MB-231 cells treated with 100 μM (**A**) and 150 μM (**B**) ORes. (**C**) Bar plot depicting KEGG pathway-based GSEA of MDA-MB-231 cells following 200 μM ORes treatment. (**D**) GSEA plots for KEGG pathways "pathways in cancer" following 200 μM ORes treatment. (**E**) Bar plot depicting KEGG pathway-based GSEA of MDA-MB-231 cells following 150 μM ORes treatment. (**F**) GSEA plots for KEGG pathways "pathways in cancer" following 150 μM ORes treatment. (**G**) GSEA plots for KEGG pathways "small cell lung cancer" following 100 μM ORes treatment.

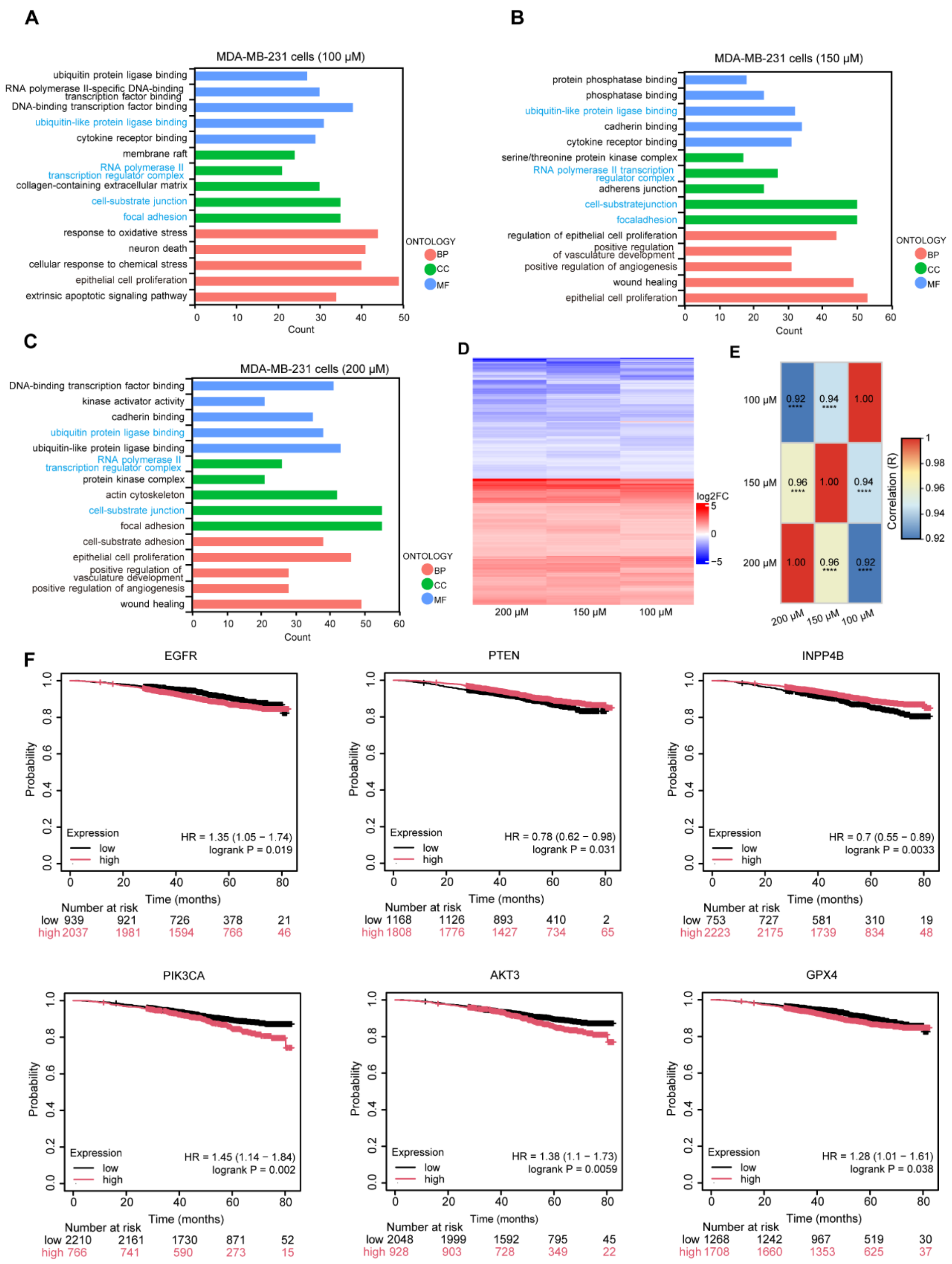

Supplementary Figure 2. DEGs in MDA-MB-231 cells treated with different concentrations ORes are enriched in tumour-related pathways. (**A-C**) GO analysis of DEGs in MDA-MB-231 cells treated with 100 μM (**A**), 150 μM (**B**), and 200 μM (**C**) ORes. MF, molecular function. CC, cellular component. BP, biological process. (**D**) Heatmap visualizing the expression of the 233 overlapping DEGs across the three treatment groups. (**E**) Pearson correlation of the three treatment groups based on the expression of 233 overlapping DEGs. (**F**) Survival curves for overall survival and in breast cancer.
